# Expenditure and financial burden for the diagnosis and treatment of colorectal cancer in China: a hospital-based, multicenter, cross-sectional survey

**DOI:** 10.1186/s40880-017-0209-4

**Published:** 2017-04-28

**Authors:** Hui-Yao Huang, Ju-Fang Shi, Lan-Wei Guo, Ya-Na Bai, Xian-Zhen Liao, Guo-Xiang Liu, A-Yan Mao, Jian-Song Ren, Xiao-Jie Sun, Xin-Yu Zhu, Le Wang, Bing-Bing Song, Ling-Bin Du, Lin Zhu, Ji-Yong Gong, Qi Zhou, Yu-Qin Liu, Rong Cao, Ling Mai, Li Lan, Xiao-Hua Sun, Ying Ren, Jin-Yi Zhou, Yuan-Zheng Wang, Xiao Qi, Pei-An Lou, Dian Shi, Ni Li, Kai Zhang, Jie He, Min Dai

**Affiliations:** 1 Program Office for Cancer Screening in Urban China, National Cancer Center/Cancer Hospital, Chinese Academy of Medical Sciences, Peking Union Medical College, 17 South Panjiayuan Lane, Chaoyang District, Beijing, 100021 P. R. China; 2 Department of Cancer Epidemiology, Henan Cancer Hospital, Affiliated Cancer Hospital of Zhengzhou University, Zhengzhou, Henan 450008 P. R. China; 3 Institute of Epidemiology and Health Statistics, Lanzhou University, Lanzhou, Gansu 730000 P. R. China; 4 Hunan Office for Cancer Control and Research, Hunan Provincial Cancer Hospital, Changsha, Hunan 410006 P. R. China; 5 Department of Health Economics, School of Health Management, Harbin Medical University, Harbin, Heilongjiang 150081 P. R. China; 6 Public Health Information Research Office, Institute of Medical Information, Chinese Academy of Medical Sciences, Beijing, 100020 P. R. China; 7 Center for Health Management and Policy, Key Lab of Health Economics and Policy, Shandong University, Jinan, Shandong 250012 P. R. China; 8 Heilongjiang Office for Cancer Control and Research, Affiliated Cancer Hospital of Harbin Medical University, Harbin, Heilongjiang 150081 P. R. China; 9 Zhejiang Office for Cancer Control and Research, Zhejiang Cancer Hospital, Hangzhou, Zhejiang 310022 P. R. China; 10 Teaching and Research Department, Affiliated Cancer Hospital of Xinjiang Medical University, Ürümqi, Xinjiang 830011 P. R. China; 11 Science and Education Department of Public Health Division, Shandong Tumor Hospital, Jinan, Shandong 250117 P. R. China; 12 Chongqing Office for Cancer Control and Research, Chongqing Cancer Hospital, Chongqing, 400030 P. R. China; 13 Cancer Epidemiology Research Center, Gansu Provincial Cancer Hospital, Lanzhou, Gansu 730050 P. R. China; 14 Department of Health Policy and Economic Research, Guangdong Provincial Institute of Public Health, Guangzhou, Guangdong 511430 P. R. China; 15 Department of Institute of Tumor Research, Henan Cancer Hospital, Affiliated Cancer Hospital of Zhengzhou University, Zhengzhou, Henan 450008 P. R. China; 16 Institute of Chronic Disease Prevention and Control, Harbin Center for Disease Control and Prevention, Harbin, Heilongjiang 150081 P. R. China; 17 Ningbo Clinical Cancer Prevention Guidance Center, Ningbo No. 2 Hospital, Ningbo, Zhejiang 315010 P. R. China; 18 Urban Office of Cancer Early Detection and Treatment, Tieling Central Hospital, Tieling, Liaoning 112000 P. R. China; 19 Institute of Chronic Non-communicable Diseases Prevention and Control, Jiangsu Provincial Center for Disease Control and Prevention, Nanjing, Jiangsu 210009 P. R. China; 20 Department of Economic Operation, Kailuan General Hospital, Tangshan, Hebei 063000 P. R. China; 21 Department of Occupational Medicine, Tangshan People’s Hospital, Tangshan, Hebei 063000 P. R. China; 22 Department of Control and Prevention of Chronic Non-communicable Diseases, Xuzhou Center for Disease Control and Prevention, Xuzhou, Jiangsu 221006 P. R. China

**Keywords:** Colorectal neoplasms, Direct expenditure, Financial burden, China

## Abstract

**Background:**

The increasing prevalence of colorectal cancer (CRC) in China and the paucity of information about relevant expenditure highlight the necessity of better understanding the financial burden and effect of CRC diagnosis and treatment. We performed a survey to quantify the direct medical and non-medical expenditure as well as the resulting financial burden of CRC patients in China.

**Methods:**

We conducted a multicenter, cross-sectional survey in 37 tertiary hospitals in 13 provinces across China between 2012 and 2014. Each enrolled patient was interviewed using a structured questionnaire. All expenditure data were inflated to the 2014 Chinese Yuan (CNY; 1 CNY = 0.163 USD). We quantified the overall expenditure and financial burden and by subgroup (hospital type, age at diagnosis, sex, education, occupation, insurance type, household income, clinical stage, pathologic type, and therapeutic regimen). We then performed generalized linear modeling to determine the factors associated with overall expenditure.

**Results:**

A total of 2356 patients with a mean age of 57.4 years were included, 57.1% of whom were men; 13.9% of patients had stage I cancer; and the average previous-year household income was 54,525 CNY. The overall average direct expenditure per patient was estimated to be 67,408 CNY, and the expenditures for stage I, II, III, and IV disease were 56,099 CNY, 59,952 CNY, 67,292 CNY, and 82,729 CNY, respectively. Non-medical expenditure accounted for 8.3% of the overall expenditure. The 1-year out-of-pocket expenditure of a newly diagnosed patient was 32,649 CNY, which accounted for 59.9% of their previous-year household income and caused 75.0% of families to suffer an unmanageable financial burden. Univariate analysis showed that financial burden and overall expenditure differed in almost all subgroups (*P* < 0.05), except for sex. Multivariate analysis showed that patients who were treated in specialized hospitals and those who were diagnosed with adenocarcinoma or diagnosed at a later stage were likely to spend more, whereas those with a lower household income and those who underwent surgery spent less (all *P* < 0.05).

**Conclusions:**

For patients in China, direct expenditure for the diagnosis and treatment of CRC seemed catastrophic, and non-medical expenditure was non-ignorable. The financial burden varied among subgroups, especially among patients with different clinical stages of disease, which suggests that, in China, CRC screening might be cost-effective.

## Background

Worldwide, colorectal cancer (CRC) is the third most commonly diagnosed cancer in men and the fourth most common in women [1]. It was estimated that, in 2012, 159,100 new male cases and 142,200 new female cases occurred in China [2]. Significant advances have been made worldwide in improving CRC patient survival, which are bound to increase the financial burden at the aggregate level, especially in the context of high prevalence and rapid population growth [1, 3]. It has been shown that patients and their families suffer both financial burden and emotional hardship [4–6]. Considerable researches have been conducted on the financial burden of cancer in the United States and other countries [7, 8]. Studies in China have been scarce, and most such studies have focused merely on the medical expenditure, as documented from hospital information systems [9].

Discerning the true financial burden helps explain the general status of a population’s health under current healthcare system, thus enabling the development of optimal policies. Furthermore, a sound understanding of the financial burden is crucial for conducting cost-effective analyses; also, it helps assess the potential expenditures and benefits of related intervention programs [10]—for example, whether the screening strategies involved in the ongoing Cancer Screening Program in Urban China (CanSPUC) are cost-effective at the current scale or an expanded scale in the future [11]. This work is of great importance in the context of limited evidence on the economic evaluation of CRC screening in China [12].

Conducted as part of the health economic evaluation research of the CanSPUC, this study aimed to estimate both medical and non-medical expenditures of overall and subgroups of CRC patients, as well as to discern the subsequent financial burdens imposed on patient families.

## Methods

### Study design and study sites

This multicenter, hospital-based, cross-sectional study was conducted between September 2012 and December 2014 in 13 study sites (Shandong, Beijing, Jiangsu, Guangdong, Zhejiang, Hebei, Liaoning, Hunan, Heilongjiang, Henan, Xinjiang, Gansu, and Chongqing). The 13 sites joined in the first 2 years after the CanSPUC startup. Thirty-seven tertiary hospitals (23 general hospitals and 14 specialized hospitals) were involved. Table 1 shows further information about the involved cities and hospitals, including population size, gross domestic product (GDP) per capita, and numbers of cities and hospitals for each site [13]. The survey was approved by the Institutional Review Board of the Cancer Hospital of the Chinese Academy of Medical Sciences. All patients provided written informed consent.

**Table 1:** Summary information and overall expenditure for diagnosis and treatment of patients with colorectal cancer in 13 study sites in China

Province	General information		Specific information on cites and hospitals involved				Overall expenditure (CNY)
	Population size in 2014^a^ (×10,000)	GDP per capita in 2014^a^ (CNY)	Number of cities	Total number of hospitals	Number of general hospitals	Number of specialized hospitals	
Shandong	9789	60,879	1 (Jinan)	1	0	1	111,813
Beijing	2152	99,995	1 (Beijing)	3	1	2	94,502
Xinjiang	2298	40,648	1 (Urumchi)	2	0	2	88,887
Hunan	6737	40,271	1 (Changsha)	6	5	1	70,168
Guangdong	10,724	58,540	5 (Five cities^b^)	2	1	1	69,238
Zhejiang	5508	73,002	2 (Hangzhou, Ningbo)	3	3	0	65,952
Heilongjiang	3833	39,226	2 (Harbin, Daqing)	1	1	0	60,245
Gansu	2591	26,433	2 (Lanzhou, Jinchang)	1	0	1	56,126
Henan	9436	37,072	1 (Zhengzhou)	6	5	1	55,829
Hebei	7384	39,984	1 (Tangshan)	1	0	1	49,332
Jiangsu	7960	81,874	2 (Nantong, Xuzhou)	1	0	1	46,181
Liaoning	4391	65,201	1 (Tieling)	9	7	2	37,103
Chongqing	2991	47,850	1 (Chongqing)	1	0	1	36,292
National total	136,782	46,652	21	37	23	14	67,408^c^

*CNY* Chinese Yuan, *GDP* gross domestic product

^a^Based on China Statistical Yearbook 2015. http://www.stats.gov.cn/tjsj/ndsj/2015/indexch.htm [13]

^b^Including Guangzhou, Shenzhen, Zhongshan, Dongguan, and Foshan

^c^The average overall expenditure for colorectal cancer diagnosis and treatment based on data from the 13 study sites in China

### Patient selection

Considering the budget from the government and previous experience, a total of 3120 CRC patients were expected for the 13 study sites. For each site, in accordance with a uniform design scheme, a stratified convenience sampling approach was used for selecting 240 clinically confirmed, primary prevalent CRC patients who were undergoing treatment in hospitals (including both newly diagnosed and existing cancer patients). To reach a sufficient power for subgroup analyses, sample sizes were balanced among cancer stages (20%–30% for each stage, from stage I to stage IV) and sex (maximum 60% for either sex). All respondents were interviewed face-to-face using a structured questionnaire at the time of discharge when most treatment expenses were incurred. Prior to the survey, we registered participation of all invited patients; also, we recorded basic information to facilitate exclusion, including age, sex, and cancer stage. For patients who were in very poor condition, family member(s) or other caregivers helped with the interview; all other interviewees were the patients themselves.

### Questionnaire contents

The questionnaire included the following five parts: (A) demographic and societal information (e.g., hospital ID, name, sex, age, education, occupation, previous-year household income, and healthcare insurance type); (B) clinical information (e.g., clinical stage, pathologic diagnosis, confirmed date, and therapeutic regimen); (C) expenditure information of the to-date whole course of illness until the survey date by clinical visit—both outpatient and inpatient, occurring both within and outside the surveyed hospitals—i.e., the start date of treatment, hospitalization duration, overall medical expenditure, overall and detailed non-medical expenditure (including additional meals, additional nutrition, transportation, accommodation, hired informal nursing, and other expenditures), predicted reimbursement ratio, and self-reported financial pressure; (D) time loss of the to-date whole course to clinical visits (both outpatient and inpatient, occurring both within and outside the surveyed hospitals)—patient working days lost and accompanying person-days of informal caregivers (relatives and friends); and (E) quality control items (e.g., investigator-evaluated reliability (excellent, good, general, or poor) of the above four parts, and signature of investigator and auditor). If the former four parts were evaluated as excellent or good reliability, the record was deemed as high quality; otherwise, it was considered as low quality.

### Estimation of expenditure and financial burden

We estimated the overall expenditure per patient for the whole course of illness, including both medical and non-medical expenditures. Medical expenditures were paid partly by the insurers; non-medical expenditures were paid entirely by the patients. We defined a newly diagnosed course as 2 months before diagnosis and 10 months after diagnosis, which is not exactly the same as the commonly used definition (1 year after diagnosis) because, in China, a large amount of money is usually spent for diagnosis before pathologic confirmation. We defined all patient-paid medical expenditure items and non-medical expenditure of a newly diagnosed course as out-of-pocket expenditure. Expenditure data presented estimates for the whole course of the illness if they were unspecified. Except when calculating the proportional breakdown of non-medical expenditures, all expenditure data were converted to the 2014 Chinese Yuan (CNY; 1 CNY = 0.163 USD) by the year-specific healthcare consumer price index of China [13].

To qualify financial pressure, we asked, “Which of the following accurately describes your family’s financial pressure from your disease?” and offered four response options: “not at all,” “somewhat but manageable,” “heavy,” and “overwhelmed.” We classified “not at all” and “somewhat but manageable” as manageable burdens; we classified the other two responses as unmanageable burdens. In addition, to objectively reflect the financial burden, we adapted the indicator of the expense-income ratio, which equals to the average out-of-pocket expense of a newly diagnosed course divided by the average previous-year household income. We used the threshold proposed by Xu et al. [14] that financial catastrophe occurs with the expense-income ratio at or exceeding 40%.

### Statistical analysis

For quality control purposes, all investigators were trained and required to check each questionnaire before ending the survey; a second research staff member would then double-check each questionnaire within 2 days of completion. All data were double-entered into EpiData 3.1 software (EpiData Association, Odense, Denmark). In addition, extensive data checking was performed using SAS 9.2 statistical software (SAS Institute, Cary/NC, USA).

SAS 9.2 statistical software was also used for data analysis. For descriptive analysis, we used percentages for qualitative variables; due to the skewed nature of quantitative variables (such as expenditure estimates), several descriptive statistics was derived as needed, including means, standard deviations, medians, and ranges. We conducted a subgroup analysis of the overall expenditure, expense-income ratio, financial pressure, and time loss by using the following variables: hospital type, age at diagnosis, sex, education, occupation, healthcare insurance type, previous-year household income, clinical stage, pathologic type, and therapeutic regimen. For the overall expenditure after logarithm transition, expense-income ratio, and time loss, a two-sample Student’s *t* test was used for a two-group comparative analysis; the analysis of variance test was used for more than two groups; and the SNK-*q* test was used for multiple comparisons. The overall expenditure of each study site was also calculated, and its spearman correlation with site-specific GDP per capita in 2014 was explored. To determine financial pressure, the Chi square test was used. To determine the influencing factors of overall expenditure, we also performed generalized linear modeling with a gamma distribution. *P* values less than 0.05 were considered statistically significant.

## Results

### Descriptive characteristics

We invited 2710 CRC patients; however, 354 (15.5%) did not participate in the survey. The main reasons for non-participation were strong refusal by patients (74.0%), followed by communication difficulties (13.0%), and strong refusal by relatives (3.4%). A total of 2356 CRC patients were finally included, with a mean age at diagnosis of 57.4 years. Of these patients, 1660 (70.5%) came from specialized hospitals; 1345 (57.1%) were men; 253 (10.7%) had college education or above; and 845 (35.9%) were farmers (Table 2). The two principal insurance types were the urban employee basic medical insurance (916 of 2356, 38.9%) and the new rural cooperative medical scheme (897 of 2356, 38.1%). The mean previous-year household income was 54,525 CNY.

**Table 2:** Characteristics of 2356 patients with colorectal cancer

Characteristic	No. of patients (%)
Hospital type	
General	696 (29.5)
Specialized	1660 (70.5)
Age at diagnosis (years)	
Mean ± SD^a^	57.4 ± 12.1
<45	361 (15.3)
45–54	542 (23.0)
55–64	787 (33.4)
≥65	666 (28.3)
Sex	
Men	1345 (57.1)
Women	1011 (42.9)
Education	
Primary school or below	727 (30.9)
Junior high school	784 (33.3)
Senior high school	592 (25.1)
Undergraduate or over	253 (10.7)
Occupation	
Farmer	845 (35.9)
Enterprise or company employee/worker	570 (24.2)
Self-employee or unemployee	354 (15.0)
Retiree	274 (11.6)
Public sector employee	259 (11.0)
Others	54 (2.3)
Previous-year household income^a,b^ (CNY)	
Mean ± SD	54,525 ± 45,822
Median (P_25_–P_75_)	40,000 (20,000–70,000)
<20,000	339 (14.9)
20,000–39,999	632 (27.7)
40,000–69,999	669 (29.4)
≥70,000	639 (28.0)
Income per patient in last 5 years^a,c^ (CNY)	
Mean ± SD	30,355 ± 29,841
Number of family members^a,d^ [median (P_25_–P_75_)]	4 (2–5)
Healthcare insurance type^e^	
Urban employee basic medical insurance	916 (38.9)
Urban resident basic medical insurance	446 (18.9)
New rural cooperative medical scheme	897 (38.1)
Commercial insurance	22 (0.9)
Self-paid	49 (2.1)
Others	24 (1.0)
Clinical stage	
I	328 (13.9)
II	630 (26.7)
III	815 (34.6)
IV	559 (23.7)
Not reported	24 (1.0)
Pathologic type	
Adenocarcinoma	2081 (88.3)
Others	176 (7.5)
Not reported	99 (4.2)
Therapeutic regimen^f^	
Surgery	886 (37.8)
Chemotherapy	784 (33.4)
Surgery and postoperative chemotherapy	333 (14.2)
Symptomatic treatment	222 (9.5)
Concurrent chemoradiotherapy	62 (2.6)
Radiotherapy	42 (1.8)
Neoadjuvant chemotherapy and surgery	16 (0.7)
Number of clinical visits^a^ [median (P_5_–P_95_)]	2 (1–5)
Number of admissions^a^ [median (P_5_–P_95_)]	1 (1–5)
Hospital stay^a,g^ (days)	
Mean ± SD	37 ± 38
Median (P_25_–P_75_)	25 (17–42)
Quality of the questionnaire	
High quality	2230 (94.7)
Low quality	126 (5.3)

*SD* standard deviation, *CNY* Chinese Yuan, *P*_*25*_ *–P*_*75*_ percentile 25 to percentile 75, *P*_*5*_ *–P*_*95*_ percentile 5 to percentile 95

^a^Except for these values, other values are presented as number of patients followed by percentage in parentheses

^b^The data of 77 patients were missing

^c^The data of 44 patients were missing

^d^The data of 24 patients were missing

^e^The data of 2 patients were missing

^f^The data of 11 patients were missing

^g^The data of 4 patients were missing

The proportions of stage I, II, III, and IV disease were 13.9, 26.7, 34.6, and 23.7%, respectively. In terms of pathologic type, most was adenocarcinoma (88.3%). Approximately one-third (37.8%) of the patients underwent surgery alone; another third (33.4%) received chemotherapy alone. The median numbers of clinical visits and admissions were 2 (percentile 5 to percentile 95 [P_5_–P_95_]: 1–5) and 1 (P_5_–P_95_: 1–5), respectively. The median hospital duration was 25 days (percentile 25 to percentile 75 [P_25_–P_75_]: 17–42 days), and the median course was 36 days (P_25_–P_75_: 12–124 days). Of the whole patient cohort, 2230 (94.7%) provided responses that were considered high quality. More information about the questionnaires is shown in Table 2.

### Overall expenditure

**Fig. 1:**
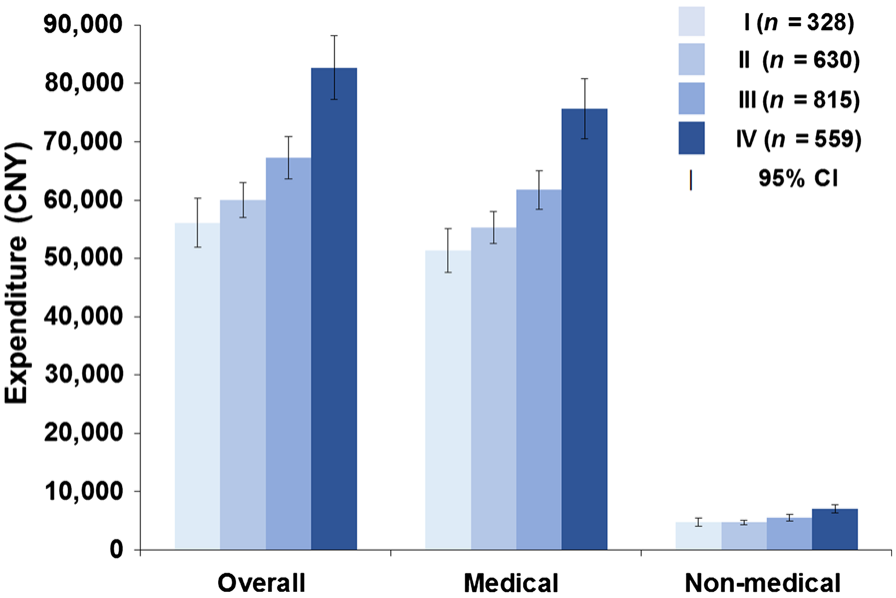
Medical and non-medical expenditures for diagnosis and treatment of patients with stage I–IV colorectal cancer. *CNY* Chinese Yuan, *CI* confidence interval. Of the 2356 patients included, 24 have no information of clinical stage

Overall mean expenditure per CRC patient was estimated to be 67,408 CNY, with 91.7% (61,829 CNY) used for medical expenditure. Overall expenditure showed a notable increase with the progression of disease (*P* < 0.001): for stages I, II, III, and IV disease, the expenditures were 56,099 CNY (95% confidence interval [CI] 51,918–60,281 CNY), 59,952 CNY (95% CI 56,971–62,932 CNY), 67,292 CNY (95% CI 63,673–70,910 CNY), and 82,729 CNY (95% CI 77,231–88,228 CNY), respectively. Multiple comparisons of overall expenditure showed that no significant difference was found between stage I and II, I and III, or II and III diseases (all *P* > 0.05); overall expenditure for stage IV disease was significantly higher than that for stages I–III diseases (*P* < 0.001). Multiple comparison of medical expenditure showed similar results; medical expenditure ranged from 51,366 CNY (95% CI 47,644–55,087 CNY) for stage I disease to 75,673 CNY (95% CI 70,551–80,794 CNY) for stage IV disease, with a 47.5% growth rate. Detailed information about medical expenditure for CRC diagnosis and treatment is shown in Fig. 1.

We found that the overall expenditure varied among different study sites (range 36,292–111,813 CNY), with Chongqing the lowest and Shandong the highest (Table 1). Spearman correlation analysis showed that the expenditure was not associated with the local economy (*r* = 0.143, *P* = 0.626). Thus, the GDP per capita was not considered in the later univariate and multivariate analyses.

Univariate analysis showed significant differences in all subgroup comparisons, except for sex (*P* = 0.181). Patients in specialized hospitals (*P* < 0.001), those who were diagnosed with adenocarcinoma (*P* < 0.001) or were diagnosed at an earlier age (*P* = 0.034), or those who were well-educated (undergraduate or higher) (*P* < 0.001) were likely to spend more compared with their control groups, whereas self-employed or unemployed patients (*P* < 0.001), underinsured patients (self-paid) (*P* = 0.007), those with a lower household income (*P* < 0.001), or who were treated with surgery (*P* < 0.001) spent less. Multivariate analysis confirmed that patients in specialized hospitals (*P* < 0.001), patients who were diagnosed with adenocarcinoma (*P* < 0.001), or patients who were diagnosed at stage IV (*P* < 0.001) were likely to spend more, whereas patients with lower household income (*P* = 0.006) or who received surgery (*P* < 0.001) spent less compared with their control groups. Detailed expenditure comparison results are shown in Tables 3 and 4.

**Table 3:** Univariate analysis of overall expenditure for diagnosis and treatment of 2356 patients with colorectal cancer

Variable	Expenditure (CNY)			Statistics^a^	*P* ^a^
	Medical	Non-medical	Overall		
Total	61,829	5579	67,408	–	–
Hospital type					
General	52,392	4893	57,285	−4.46	<0.001
Specialized	65,786	5866	71,652		
Age at diagnosis (years)					
<45	65,887	6122	72,009	2.90	0.034
45–54	66,303	6049	72,352		
55–64	59,416	5328	64,744		
≥65	58,840	5198	64,038		
Sex					
Men	62,526	5751	68,277	1.34	0.181
Women	60,903	5350	66,253		
Education					
Primary school or below	57,354	4883	62,237	8.36	<0.001
Junior high school	58,898	5191	64,089		
Senior high school	65,095	5805	70,900		
Undergraduate or higher	76,132	8250	84,382		
Occupation					
Farmer	57,796	4973	62,769	8.19	<0.001
Enterprise or company employee/worker	63,563	5859	69,422		
Self-employee or unemployee	54,449	4640	59,089		
Retiree	70,629	7835	78,464		
Public sector employee	72,707	5628	78,335		
Others	58,197	6579	64,776		
Healthcare insurance type					
Urban employee basic medical insurance	66,458	6289	72,747	3.17	0.007
Urban resident basic medical insurance	60,118	4861	64,979		
New rural cooperative medical scheme	58,046	5062	63,108		
Commercial insurance	61,233	2958	64,191		
Self-paid	53,914	5855	59,769		
Others	78,293	13,023	91,316		
Previous-year household income (CNY)					
<20,000	53,581	4570	58,151	5.75	<0.001
20,000–39,999	61,461	5193	66,654		
40,000–69,999	62,972	5586	68,558		
≥70,000	65,371	6188	71,559		
Pathologic type					
Adenocarcinoma	62,759	5636	68,395	3.35	<0.001
Others	53,279	4501	57,780		
Therapeutic regimen					
Surgery	51,759	3812	55,571	14.04	<0.001
Chemotherapy	68,749	6818	75,567		
Surgery and postoperative chemotherapy	63,068	5702	68,770		
Symptomatic treatment	55,970	5237	61,207		
Concurrent chemoradiotherapy	114,491	12,944	127,435		
Radiotherapy	88,857	7989	96,846		
Neoadjuvant chemotherapy and surgery	71,557	9682	81,239		

*CNY* Chinese Yuan

^a^Two-sample Student’s *t* test after logarithm transition was used for binary classification variables, including hospital type, sex, and pathologic type; analysis of variance test after logarithm transition was used for other multiple categorical variables, including age at diagnosis, education, occupation, insurance type, household income, and therapeutic regimen

**Table 4:** Multivariate analysis of overall expenditure for diagnosis and treatment of 2356 patients with colorectal cancer

Characteristic	Estimate (95% CI)	*P*
Intercept	10.4 (10.2, 10.5)	<0.001
Hospital type (Ref = general)		
Specialized	0.2 (0.1, 0.2)	<0.001
Age at diagnosis (years) (Ref = ≥65)		
<45	0.0 (−0.1, 0.1)	0.419
45–54	0.1 (0.0, 0.2)	0.131
55–64	0.0 (−0.1, 0.1)	0.522
Sex (Ref = women)		
Men	0.0 (−0.1, 0.1)	0.969
Education (Ref = primary school or below)		
Junior high school	0.0 (−0.1, 0.1)	0.815
Senior high school	0.0 (0.0, 0.1)	0.310
Undergraduate or higher	0.1 (0, 0.2)	0.134
Occupation (Ref = self-employee or unemployee)		
Farmer	0.0 (−0.1, 0.2)	0.493
Enterprise or company employee/worker	−0.1 (−0.2, 0.0)	0.057
Retiree	0.1 (−0.1, 0.2)	0.377
Public sector employee	0.2 (0.1, 0.3)	0.004
Other	0.0 (−0.3, 0.2)	0.778
Healthcare insurance type (Ref = new rural cooperative medical scheme)		
Urban employee basic medical insurance	0.0 (−0.1, 0.1)	0.641
Urban resident basic medical insurance	0.0 (−0.1, 0.1)	0.707
Commercial insurance	0.0 (−0.3, 0.4)	0.792
Self-paid	0.0 (−0.2, 0.2)	0.905
Other	0.4 (0.1, 0.7)	0.020
Previous-year household income (CNY) (Ref = ≤20,000)		
20,000–39,999	0.1 (0.0, 0.2)	0.006
40,000–69,999	0.1 (0.0, 0.2)	0.046
≥70,000	0.1 (0.0, 0.2)	0.006
Clinical stage (Ref = I)		
II	0.0 (−0.1, 0.1)	0.822
III	0.1 (0.0, 0.2)	0.099
IV	0.3 (0.1, 0.4)	<0.001
Pathologic type (Ref = others)		
Adenocarcinoma	0.2 (0.1, 0.3)	<0.001
Therapeutic regimen (Ref = surgery)		
Chemotherapy	0.2 (0.1, 0.3)	<0.001
Surgery and postoperative chemotherapy	0.2 (0.1, 0.3)	<0.001
Symptomatic treatment	0.1 (0.0, 0.2)	0.170
Concurrent chemoradiotherapy	0.8 (0.6, 1.0)	<0.001
Radiotherapy	0.5 (0.3, 0.7)	<0.001
Neoadjuvant chemotherapy and surgery	0.2 (−0.2, 0.6)	0.251

*CNY* Chinese Yuan, *CI* confidence interval

### Non-medical expenditure

**Fig. 2:**
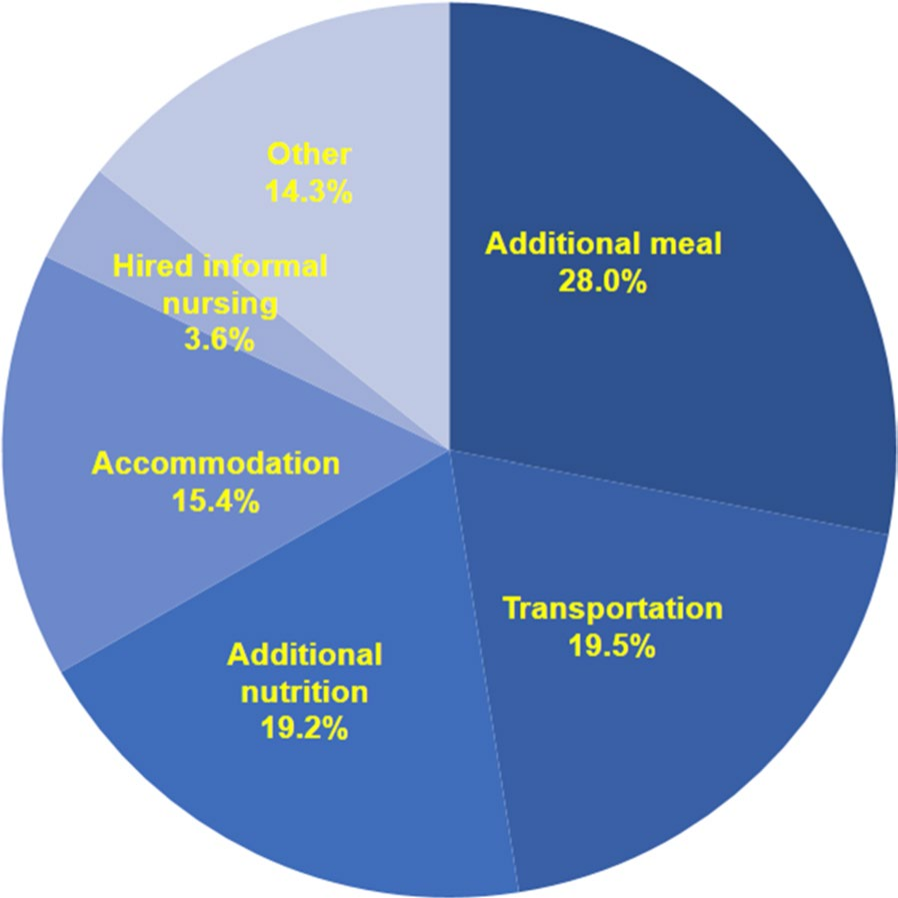
Proportional breakdown of non-medical expenditures for diagnosis and treatment of colorectal cancer

Non-medical expenditure accounted for 8.3% of the overall expenditure (5588 CNY per CRC patient). Additional meal contributed the largest proportion (1566 CNY, 28.0%), followed by transportation (1089 CNY, 19.5%) and additional nutrition (1075 CNY, 19.2%). Multiple comparisons of non-medical expenditure between CRC patients stage I-IV disease showed differences between all the two subgroups except stages II and III. Figures 1 and 2 show more detailed information about non-medical expenditure.

### Financial burden

As a whole, the overall expenditure of a newly diagnosed illness course was 58,778 CNY, accounting for 87.2% of that of the to-date whole course of illness. With the predicted reimbursement ratio equaling 46.5%, out-of-pocket expenditure amounted to 32,649 CNY, accounting for 59.9% of the previous-year household income. That made 75.0% of the families perceive an unmanageable burden (47.4% heavy, 27.6% overwhelmed); only 18.3% perceived a somewhat but manageable burden, and 6.7% perceived no burden at all. The influencing factors associated with the expense-income ratio and the proportion of families with an unmanageable burden coincide with each other quite well; moreover, these factors were also allied with the overall expenditure of CRC patients in China.

Compared with patients from general hospitals, patients from specialized hospitals tended to have a higher expense-income ratio (0.684 vs. 0.432, *P* < 0.001) and more families with unmanageable burden (76.4% vs. 71.4%, *P* < 0.001). Similarly, patients who were diagnosed at an earlier age (younger than 45 years) were likely to expend more than those diagnosed at older age (*P* = 0.019); among these patients, 79.6% experienced an unmanageable burden. The expense-income ratio for patients with poor education (i.e., primary school or less) was 0.769, making 84.0% of them experience an unmanageable burden, which was much higher than that of well-educated patients (*P* < 0.001). The gap was even apparent in terms of the household income: those with lower household income (i.e., less than 20,000 CNY) spent a larger share of the household income for CRC diagnosis and treatment, and more families felt stressed (*P* < 0.001). Those with income lower than 20,000 CNY spent more than three times their household income for CRC diagnosis and treatment, making 92.3% of these families unable to afford treatment. In terms of therapeutic regimen, the expense-income ratios of patients who received symptomatic treatment and those who received neoadjuvant chemotherapy and surgery were the lowest and highest (0.489 vs. 1.192, *P* < 0.001); the proportion of families who perceived an unmanageable burden was lowest for patients who received symptomatic treatment and highest for those who received radiotherapy (69.0% vs. 85.7%, *P* < 0.001). Although the expense-income ratio was similar among patients with CRC of various stages (*P* = 0.054), we still found that families of stage IV CRC patients suffered the highest pressure (27.8% heavy, 50.9% overwhelmed; *P* < 0.001). As expected, the expense-income ratio of farmers was the highest (0.977, *P* < 0.001): 90.1% of farmer families found treatment expenses unmanageable. However, patients who had new rural cooperative medical scheme insurance faced a similar dilemma and spent 85.8% of their household income, resulting in 88.6% of these families experiencing unmanageable financial burden. Neither the expense-income ratio nor financial pressure was statistically sensitive to sex (*P* = 0.053) or pathologic type (*P* = 0.083). More information about the financial burden of overall expenditure is shown in Table 5.

**Table 5:** Financial effect of overall expenditure on colorectal cancer patient’s family

Characteristic	Expenditure of a newly diagnosed disease course^a^ (CNY)	Self-reported predicted reimbursement ratio^b^ (%)	Out-of-pocket expenditure^b,c^ (CNY) (A)	Previous-year household income (CNY) (B)	Expense-income ratio (A/B)		Self-reported financial pressure^e^ [*n* (%)]				
					Value	*P* ^d^	Not at all	Somewhat but manageable	Heavy	Overwhelmed	*P* ^f^
Total	58,778	46.5	32,649	54,525	0.599	–	158 (6.7)	429 (18.3)	647 (27.6)	1111 (47.4)	–
Hospital type											
General	48,258	50.2	26,742	61,866	0.432	<0.001	70 (10.1)	128 (18.5)	200 (28.9)	294 (42.5)	<0.001
Specialized	63,189	44.9	35,154	51,403	0.684		88 (5.3)	301 (18.2)	447 (27.0)	4817 (9.4)	
Age at diagnosis (years)											
<45	59,930	41.3	36,670	56,888	0.645	0.019	12 (3.4)	61 (17.0)	75 (20.9)	210 (58.7)	<0.001
45–54	62,643	46.8	34,621	57,088	0.606		34 (6.3)	86 (16.0)	142 (26.3)	277 (51.4)	
55–64	58,340	45.8	32,173	51,441	0.625		55 (7.0)	133 (16.9)	228 (29.0)	369 (47.0)	
≥65	55,527	49.8	29,425	54,792	0.537		57 (8.6)	149 (22.5)	202 (30.5)	255 (38.5)	
Sex											
Men	59,496	47.8	32,180	55,654	0.578	0.293	85 (6.4)	256 (19.2)	390 (29.2)	605 (45.3)	0.053
Women	57,823	44.7	33,273	52,999	0.628		73 (7.2)	173 (17.1)	257 (25.5)	506 (50.1)	
Education											
Primary school or below	56,196	40.8	33,563	43,647	0.769	<0.001	28 (3.9)	88 (12.1)	206 (28.4)	404 (55.6)	<0.001
Junior high school	55,499	44.7	33,151	51,301	0.646		52 (6.7)	130 (16.7)	207 (26.6)	389 (50.0)	
Senior high school	62,019	50.1	31,598	62,863	0.503		44 (7.5)	136 (23.1)	172 (29.3)	236 (40.1)	
Undergraduate or higher	68,776	59.8	30,943	75,844	0.408		34 (13.4)	75 (29.6)	62 (24.5)	82 (32.4)	
Occupation											
Farmer	56,812	37.0	36,961	37,845	0.977	<0.001	11 (1.3)	72 (8.6)	214 (25.4)	544 (64.7)	<0.001
Enterprise or company employee/worker	59,787	56.7	28,593	60,396	0.473		53 (9.3)	123 (21.7)	166 (29.3)	225 (39.7)	
Self-employed or unemployee	51,594	35.1	33,266	57,242	0.581		23 (6.6)	48 (13.7)	93 (26.5)	187 (53.3)	
Retiree	64,059	60.3	28,342	69,155	0.410		27 (9.9)	89 (32.5)	82 (29.9)	76 (27.7)	
Public sector employee	66,992	58.8	29,102	72,026	0.404		34 (13.2)	84 (32.6)	82 (31.8)	58 (22.5)	
Other	59,806	31.1	42,908	74,245	0.578		10 (18.5)	13 (24.1)	10 (18.5)	21 (38.9)	
Healthcare insurance type											
Urban employee basic medical insurance	62,021	58.8	28,021	65,258	0.429	<0.001	108 (11.8)	222 (24.3)	275 (30.1)	308 (33.7)	<0.001
Urban resident basic medical insurance	56,065	49.1	29,482	50,466	0.584		18 (4.0)	110 (24.7)	116 (26.1)	201 (45.2)	
New rural cooperative medical scheme	56,880	35.1	37,684	43,898	0.858		20 (2.2)	81 (9.1)	235 (26.3)	556 (62.3)	
Commercial insurance	59,424	25.2	42,769	60,273	0.710		4 (18.2)	4 (4.5)	1 (22.7)	5 (54.5)	
Self-paid	47,771	0.0	47,771	79,956	0.597		5 (10.2)	5 (18.4)	9 (18.4)	9 (53.1)	
Other	80,807	64.9	39,869	57,855	0.689		3 (12.5)	6 (25.0)	7 (29.2)	8 (33.3)	
Previous-year household income (CNY)											
<20,000	51,323	37.6	33,207	10,258	3.237	<0.001	6 (1.8)	20 (6.0)	61 (18.2)	249 (74.1)	<0.001
20,000–39,999	58,978	44.8	34,618	26,072	1.328		34 (5.4)	66 (10.5)	160 (25.4)	371 (58.8)	
40,000–69,999	58,885	49.4	30,979	49,932	0.620		41 (6.2)	155 (23.3)	198 (29.8)	271 (40.8)	
≥70,000	62,086	50.0	31,769	110,959	0.286		68 (10.7)	179 (28.1)	207 (32.5)	182 (28.6)	
Clinical stage											
I	50,425	50.0	26,950	48,454	0.556	0.054	49 (15.0)	64 (19.6)	79 (24.2)	135 (41.3)	<0.001
II	56,097	45.9	31,305	53,801	0.582		38 (6.1)	133 (21.2)	176 (28.1)	280 (44.7)	
III	59,782	45.0	32,969	53,401	0.617		43 (5.3)	139 (17.1)	233 (28.7)	398 (49.0)	
IV	65,209	47.7	36,610	61,333	0.597		27 (4.9)	91 (16.4)	154 (27.8)	282 (50.9)	
Pathologic type											
Adenocarcinoma	59,653	46.9	32,756	55,065	0.595	0.139	142 (6.9)	373 (18.0)	565 (27.3)	990 (47.8)	0.083
Other	52,955	44.8	31,272	51,593	0.606		12 (6.8)	38 (21.6)	59 (33.5)	67 (38.1)	
Type of therapy											
Surgery	53,083	45.2	29,521	48,047	0.614	<0.001	82 (9.3)	172 (19.5)	262 (29.7)	367 (41.6)	<0.001
Chemotherapy	62,476	47.9	34,846	61,024	0.571		43 (5.5)	123 (15.8)	205 (26.3)	409 (52.4)	
Surgery and postoperative chemotherapy	62,217	48.9	33,251	54,218	0.613		8 (2.4)	63 (18.9)	95 (28.5)	167 (50.2)	
Symptomatic treatment	47,408	41.3	29,608	60,538	0.489		22 (10.0)	46 (21.0)	51 (23.3)	100 (45.7)	
Concurrent chemoradiotherapy	97,958	52.8	46,001	46,232	0.995		2 (3.2)	13 (21.0)	17 (27.4)	30 (48.4)	
Radiotherapy	79,945	42.0	46,824	56,359	0.831		0 (0.0)	6 (14.3)	10 (23.8)	26 (61.9)	
Neoadjuvant chemotherapy and surgery	77,269	48.1	43,154	36,200	1.192		0 (0.0)	5 (31.3)	4 (25.0)	7 (43.8)	

*CNY* Chinese Yuan

^a^Two months before and ten months after diagnosis

^b^The data of 12 patients were missing

^c^Out-of-pocket expenditure = [∑(1 − self-reported predicted reimbursement ratio) × medical expenditure of a newly diagnosed course + non-medical expenditure of a newly diagnosed course]/n; n refers to the sample size

^d^Two-sample Student’s *t* test after logarithm transition was used for binary classification variables, including hospital type, sex, and pathologic type; analysis of variance test after logarithm transition was used for other multiple categorical variables, including age at diagnosis, education, occupation, insurance type, household income, and therapeutic regimen

^e^The data of 11 patients were missing for hospital type, age at diagnosis, sex, education, occupation and insurance type; the data of 21, 35, 88 and 110 patients were missing for type of therapy, clinical stage, household income and pathologic type, respectively

^f^We classified “not at all” and “somewhat but manageable” as manageable burdens and the other two responses as unmanageable burdens. The Chi square test was used for subgroup comparisons

### Time loss

Mean overall time loss amounted to 95.9 person-days—54.0 person-days (56.3%) for patients and 41.9 person-days (43.7%) for caregivers. If crudely converted by the 2014 minimum monthly wage of 1560 CNY in Beijing [15], mean wage loss amounted to 6652 CNY. Patients from specialized hospitals (*P* < 0.001) or those who were diagnosed with adenocarcinoma (*P* = 0.026) suffered relatively more time loss than patients from general hospitals or those who were diagnosed with other pathologic types; conversely, patients who were self-employed or unemployed (*P* < 0.001), covered by the Urban Resident Basic Medical Insurance or underinsured (*P* = 0.003), diagnosed with stage I–II disease (*P* < 0.001), or underwent surgery (*P* < 0.001) suffered less than their corresponding control groups. Nevertheless, when stratified by age at diagnosis (*P* = 0.516), sex (*P* = 0.191), education (*P* = 0.138), or household income (*P* = 0.219), no difference was observed. Detailed findings for time loss are shown in Table 6.

**Table 6:** Time loss due to colorectal cancer diagnosis and treatment

Characteristic	Time loss (person-days)^a^			Statistics^b^	*P* ^b^
	Overall	Patients	Caregivers		
Total	95.9	54.0	41.9	–	–
Hospital type					
General	40.0	38.7	78.6	−4.10	<0.001
Specialized	59.9	43.3	103.2		
Age at diagnosis (years)					
<45	103.5	61.7	41.7	0.76	0.516
45–54	98.3	55.6	42.7		
55–64	95.4	53.6	41.8		
≥65	90.7	49.0	41.6		
Sex					
Men	98.8	55.7	43.1	1.31	0.191
Women	92.2	51.8	40.3		
Education					
Primary school or below	95.4	53.7	41.7	1.84	0.138
Junior high school	94.9	54.3	40.6		
Senior high school	95.2	52.0	43.2		
Undergraduate or higher	102.6	58.7	43.9		
Occupation					
Farmer	101.1	58.1	43.0	4.80	<0.001
Enterprise or company employee/worker	98.8	53.7	45.1		
Self-employee or unemployee ununemployee	78.8	45.8	33.0		
Retiree	93.5	52.7	40.8		
Public sector employee	98.6	53.8	44.8		
Others	96.5	52.9	43.6		
Healthcare insurance type					
Urban employee basic medical insurance	99.7	54.6	45.0	3.64	0.003
Urban resident basic medical insurance	82.2	43.3	38.9		
New rural cooperative medical scheme	99.3	58.8	40.4		
Commercial insurance	87.0	60.4	26.5		
Self-paid	85.0	46.2	38.8		
Other	127.0	65.0	62.0		
Previous-year household income (CNY)					
<20,000	98.1	56.1	42.0	1.48	0.219
20,000–39,999	99.3	57.3	42.0		
40,000–69,999	96.7	54.5	42.2		
≥70,000	92.1	50.3	41.8		
Clinical stage					
I	76.1	40.1	36.0	16.05	<0.001
II	77.4	42.8	34.6		
III	98.0	55.6	42.4		
IV	126.8	73.7	53.1		
Pathologic type					
Adenocarcinoma	97.0	55.0	42.0	2.25	0.026
Others	82.4	46.6	35.8		
Therapeutic regimen					
Surgery	70.6	39.9	30.7	18.28	<0.001
Chemotherapy	115.7	66.1	49.6		
Surgery and postoperative chemotherapy	98.2	55.8	42.5		
Symptomatic treatment	93.3	49.2	44.1		
Concurrent chemoradiotherapy	196.2	103.0	93.2		
Radiotherapy	188.0	105.1	82.9		
Neoadjuvant chemotherapy and surgery	91.8	53.7	38.1		

*CNY* Chinese Yuan

^a^The data of 89 patients were missing

^b^Two-sample Student’s *t* test after logarithm transition was used for binary classification variables, including hospital type, sex, and pathologic type; analysis of variance test after logarithm transition was used for other multiple categorical variables, including age at diagnosis, education, occupation, insurance type, household income, and therapeutic regimen

## Discussion

Our study provided much-needed data on direct medical and non-medical expenditures associated with prevalent CRC and the resulting financial burden. We found that direct expenditure was catastrophic and burdensome and varied greatly among different subgroups.

In our study, we found that the mean direct expenditure per CRC patient was 67,408 CNY. According to a recent review of the financial burden of CRC in China, only one study included both medical and non-medical expenditures per patient; others focused only on medical expenditure [9]. The earliest study was reported in 1999, and the most recent was in 2014 [9]. Except for one study (~50,000 CNY), all others showed expenses amounting to less than half of that in our study (61,829 CNY), mainly because of their relatively short course and our uncovering of expenses outside the surveyed hospitals [9]. According to the latest data published in *Lancet*, CRC was the most costly cancer among the six most common cancers in urban China [16]. Compared internationally, the absolute quantity of overall expenditure in China was much less than that in the United States and Canada [17, 18]. The ratio of the expenditure in annual GDP per capita was 1.4 and 0.6 in our survey, whereas that for both the United States and Canada was lower than 1.0 [19].

We found that several variables were significant for overall expenditure, including hospital type, occupation, household income, clinical stage, pathologic type, and therapeutic regimen. Notably, in line with a recent research [20] and review [21], we found that expenditure was higher for patients with late stage (stage III and IV) CRC than for those with early stage (stage I and II) CRC, which may be linked to longer hospitalization stays and more expensive treatments, such as targeted biological therapies. In contrast, patients with stage I disease mostly underwent surgery and spent much less. These findings suggest the potential cost-effectiveness of early detection and treatment. Although we attempted to balance stage-specific cases, only 13.9% of all cases were stage I, which reflects the lack of early diagnosis and treatment. However, under the resource and financial constraints in China, policymakers require more evidence of cost-effectiveness before expanding the scope of CRC screening.

Additionally, we found that non-medical expenditure for the diagnosis and treatment of CRC was a significant component, reaching 8.3% of the overall expenditure. It was higher than that found in the only previous relevant study in China (5.4%) [22]. Of the 55 overseas studies on financial burden, only nine investigated non-medical expenditure. The detailed non-medical expenditure were mainly comprising the wage losses of both caregivers and patients [23], which was different from those reported by Drummond et al. [24] and Cheng et al. [25], as well as those reported in our study.

Regarding time loss, CRC diagnosis and treatment caused an average wage loss of 1560 CNY, accounting for 3.3% of GDP per capita of China in 2014, which was substantially underestimated. The ratio of wage loss in annual GDP per capita for the only previous study in China was 12.2% [22], whereas those found in studies in the United States and in Canada were 25.6% [26] and 289.5% [27], respectively. This substantial gap could be partly explained by the low labor cost and special healthcare delivery model in China and potentially by methodological differences. Numeric differences exist among these studies, but it is more important to observe the consistent conclusions and directions. The time loss of both patients and caregivers was a considerable component of the financial burden, and more attention was recently paid to time cost and productivity cost [28–30]. However, in China, these components have been rarely calculated previously [9].

Obviously, much work remains to determine the comprehensive expenditure, especially including direct non-medical expenditure, indirect expenditure, and intangible expenditure. Moreover, in China, no attention has been given to lifetime expenditure or specifics such as treatment phase (initial, continuing, or terminal). In studies of financial burden, opportunities and challenges coexist.

Concerning financial burden, patients spent 59.9% of their household income for one year of CRC diagnosis and treatment, and 75.0% of the families perceived an unmanageable financial burden. In contrast, in the United States, 25.0% of insured patients spent approximately one-third of their annual income on healthcare, and 39.9% spent approximately one-fifth of their annual income [30]. In Canada, the proportion of patients who perceived an unmanageable financial burden was only 3.9%; even including those who perceived a significant but manageable burden, it came to only 20.4% [31], which was much lower than that in our study. Thus, we can surmise that the plight of CRC patients and their families in China is worse than that of CRC patients in the United States and Canada. Heterogeneity in terms of data source or methodology occasionally makes it difficult to compare across studies; nevertheless, such a large gap does deserve the government’s attention. Developing corresponding policies to control out-of-pocket expenses can help patients and their families in China better cope with serious diseases such as cancer.

Several key influencing factors for the expense-income ratio and the proportion of families perceiving an unmanageable financial burden were found to be similar to those for overall expenditure, which perhaps was not coincidental when we combined evidence reported elsewhere [32–34]. Notably, patients in the lowest household income group were in the worst financial situation, although the absolute quantity of expenditure was the least. Not surprisingly, the inability to pay prevents them from receiving sufficient healthcare [35]. Well-educated patients generally had higher incomes and spent more but were less stressed compared with their control groups. Because farmers generally had new rural cooperative medical scheme insurance and lower income, it seems self-evident that their direct expenditure was the lowest, and vice versa for public sector employees or retirees. These differences suggest that, in keeping with principles of justice and fairness, health service delivery reforms, such as to the medical insurance system, should consider providing more assistance to vulnerable populations.

This survey had several limitations. First, recall bias may have resulted from the retrospective nature of the questionnaire survey. Second, selection bias may have resulted from high-level hospitals and non-random sampling, although in China CRC patients are almost exclusively treated in tertiary hospitals. Third, the expenditure may have been underestimated because it covered only the to-date whole course. Another issue is risk factors for high expenditure; further multiple factor analysis is needed. Finally, although we could determine the potential effect of wage loss on perceived financial pressure, we considered only medical and non-medical expenditures.

## Conclusions

Research on calculating the financial burden of CRC in China is still in its initial phase, and more is needed. We found that, for patients in China, expenditure for diagnosis and treatment of CRC seemed catastrophic, and non-medical expenditure cannot be ignored. Expenditure and financial burden varied among subgroups, especially for patients with disease of different clinical stages, suggesting that, in China, CRC screening might be cost-effective. Our findings also support the policy of medical insurance and payment system reform for affordable and equitable access to quality healthcare, which should be considered before further research on comprehensive expenditure is done.

## Abbreviations

CRC: colorectal cancer
CanSPUC: Cancer Screening Program in Urban China
CNY: Chinese Yuan
